# Autophagic cell death associated to Sorafenib in renal cell carcinoma is mediated through Akt inhibition in an ERK1/2 independent fashion

**DOI:** 10.1371/journal.pone.0200878

**Published:** 2018-07-26

**Authors:** Leticia Serrano-Oviedo, Marta Ortega-Muelas, Jesús García-Cano, María Ll. Valero, Francisco J. Cimas, Raquel Pascual-Serra, Diego M. Fernandez-Aroca, Olga Roche, María J. Ruiz-Hidalgo, Borja Belandia, José M. Giménez-Bachs, Antonio S. Salinas, Ricardo Sanchez-Prieto

**Affiliations:** 1 Laboratorio de Oncología, Unidad de Medicina Molecular, Centro Regional de Investigaciones Biomédicas Universidad de Castilla-La Mancha, Unidad Asociada de Biomedicina UCLM, Unidad asociada al CSIC, Albacete, Spain; 2 Departamento de ciencias Médicas, Facultad de Medicina, Universidad de Castilla-La Mancha, Albacete, Spain; 3 Área de Bioquímica y Biología Molecular, Facultad de Medicina, Universidad de Castilla-La Mancha, Albacete, Spain; 4 Departamento de Biología del Cáncer, Instituto de investigaciones Biomedicas Alberto Sols (CSIC-UAM), Unidad Asociada de Biomedicina UCLM, Unidad asociada al CSIC, Madrid, Spain; 5 Servicio de Urologia, Unidad de investigación, Complejo Hospitalario de Albacete, Facultad de Medicina, Albacete, Spain; Univerzitet u Beogradu, SERBIA

## Abstract

**Objectives:**

To fully clarify the role of Mitogen Activated Protein Kinase in the therapeutic response to Sorafenib in Renal Cell Carcinoma as well as the cell death mechanism associated to this kinase inhibitor, we have evaluated the implication of several Mitogen Activated Protein Kinases in Renal Cell Carcinoma-derived cell lines.

**Materials and methods:**

An experimental model of Renal Cell Carcinoma-derived cell lines (ACHN and 786-O cells) was evaluated in terms of viability by MTT assay, induction of apoptosis by caspase 3/7 activity, autophagy induction by LC3 lipidation, and p62 degradation and kinase activity using phospho-targeted antibodies. Knock down of ATG5 and ERK5 was performed using lentiviral vector coding specific shRNA

**Results:**

Our data discard Extracellular Regulated Kinase 1/2 and 5 as well as p38 Mitogen Activated Protein Kinase pathways as mediators of Sorafenib toxic effect but instead indicate that the inhibitory effect is exerted through the PI3K/Akt signalling pathway. Furthermore, we demonstrate that inhibition of Akt mediates cell death associated to Sorafenib without caspase activation, and this is consistent with the induction of autophagy, as indicated by the use of pharmacological and genetic approaches.

**Conclusion:**

The present report demonstrates that Sorafenib exerts its toxic effect through the induction of autophagy in an Akt-dependent fashion without the implication of Mitogen Activated Protein Kinase. Therefore, our data discard the use of inhibitors of the RAF-MEK-ERK1/2 signalling pathway in RCC and support the use of pro-autophagic compounds, opening new therapeutic opportunities for Renal Cell Carcinoma.

## Introduction

Cancer therapy has evolved from conventional chemotherapy, targeting general molecules/processes with key roles in cellular homeostasis (e.g. DNA damage response, cell cycle etc.), to a more specific therapy based on molecular alterations exclusively present in tumor cells, the first example being Imatibinib [1]. Since then, the list of compounds targeting protein kinases and signalling pathways is increasing exponentially. Among them, Sorafenib (BAY-43-9006) has become one of the best and more studied examples of targeted therapies. Discovered initially as an inhibitor of RAF kinase [2], it was first used as an antitumor agent in melanomas with disappointing results (for a review see [3]. However, later it was shown to have a potent inhibitory effect on the tyrosine kinase activity of receptors such as VEGFR1/3 and PDGR [4], allowing its use in several pathologies including Hepatocellular Carcinoma, Thyroid Carcinoma and Renal Cell Carcinoma (RCC) (for a review see [5]. Regarding RCC, the molecular basis of Sorafenib-based therapy is not fully understood, but it seems to be linked to the effect exerted on VEGF and PDGF receptors. Interestingly, the natural ligands of these receptors are controlled by the VHL-HIF system, the hallmark of the most common subtype of RCC (for a review see [6]). Indeed, other tyrosine kinase inhibitors of VEGFR and PDGFR, such as Sunitinib [7], are currently used in the treatment of RCC [8].

The classical Mitogen Activated Protein Kinase (MAPK) family is composed of four large groups of kinases that have been extensively implicated in human pathology (for a review see [9]). Probably the best studied MAPK group in cancer, due to its ability in promoting cell growth, is the ERK1/2 subfamily. Interestingly, almost all components of this signalling pathway have been considered as potential targets in cancer therapy (for a review see [10] althoughtheir role still need to be fully elucidated [11]. Nonetheless, this signalling pathway has been related to the therapeutic effectiveness of several tyrosine kinase inhibitors, such as Imatinib [12], Genfitinib [13], Erlotinib [14] Sunitinib [15], and even Sorafenib [16]. Interestingly, VEGFR and PDGFR signalling pathways, two main targets of Sorafenib in RCC, have been connected with MAPK since the late 90’s [17–19]. However, the role of ERK1/2 in the response to Sorafenib in RCC has been studied only in co-culturing resistant models for Sorafenib or Sunitinib [20,21], and no definitive conclusion about the role of MAPK in the primary response or in *de novo* resistance to Sorafenib in RCC has been established so far.

In an attempt to fully clarify the role of MAPKs in the response of RCC-derived cell lines to Sorafenib, we have studied the implication of different MAPKs in the cell death mechanism triggered by Sorafenib. Our data clearly show that caspase-independent cell death associated to Sorafenib is consistent with autophagy, and it is mediated through the inhibition of the PI3/Akt signalling pathway in an ERK1/2-independent fashion. Therefore, our data are a proof of concept for the rational use of proautophagic compounds, such as mTOR inhibitors, in RCC therapy.

## Materials and methods

### Cell lines and plasmids

ACHN cells (ATCC) were cultured in Eagle’s Minimum Essential medium (EMEM), and 786-O cells (ATCC) were cultured in Dulbecco’s Modified Eagle’s medium (DMEM) with 1% non-essential amino acids. Both cell lines were supplemented with 10% fetal bovine serum and 1% glutamine plus antibiotics (Penicillin, Streptomycin and Amphotericin B). All cell culture reagents were provided by Sigma Aldrich (Tres Cantos, Madrid, Spain). Cells were maintained at 5% CO_2_ and 37°C.

### Chemicals and antibodies

Antibodies against active ERK1/2 (#4377), p38 MAPK (#9215), Akt (#9916), mTOR active (ser 2448. #5536), total ERK5 (#3372), mTOR (#2972), and ATG5 (#2630) were purchased from Cell Signalling Technologies (Izasa, Madrid, Spain). Antibodies against p62/SQTM1 (sc-28359), total p38α (sc-535), total ERK2 (sc-154), total Akt (sc-8312), and α-Tubulin (sc-32293) were from Santa Cruz Biotechnology (Quimigen, Madrid, Spain). Antibodies against LC3 and Vinculin were purchased from Sigma-Aldrich (Tres cantos, Madrid, Spain). Sorafenib, MK-2206 (S1078), U0126 (S1102), PD98059, and SB203580 were purchased from Selleckchem (Deltaclon, Madrid, Spain). 3-methyladenine (3MA) (189490) was purchased from Calbiochem/Merck—Millipore (Madrid, Spain). These chemicals were diluted in DMSO and stored at -80°C until use.

### Viability assays

Sub-confluent monolayer cultures were trypsinized, and an initial population of 2×10^4^ cells/well was seeded in 24-well plates. Twenty-four hours later, media were discarded and replaced by media containing either drugs, inhibitors, or both at concentrations indicated in each case. After treatment, cell proliferation was analysed at 48 hours by an MTT-based assay as previously described [22]. Briefly, MTT reactant (Thiazolyl Blue Tetrazolium Bromide, M2128, Sigma Aldrich) at 5 mg/ml in a PBS solution, was added to the cells in a 1:10 ratio (MTT solution/culture medium] and incubated during 1 hour at 37°C. Then, media were discarded and the formazan crystals formed inside the cells were recovered with DMSO. Subsequently, the optical density at 570 nm was evaluated to quantify the amount of formazan crystals, which is proportional to the number of viable cells. Viability was compared to untreated controls (100%).

### Caspase assays

For caspase activation assays, cells were plated at a density of 10^4^ cells/well in opaque 96-well plates 24 hours prior to treatment. Twenty-four hours after treatment, activation of effector caspases 3 and 7 was evaluated with Promega’s CaspaseGlo kit (G8090) following manufacturer’s instructions. Resulting mixtures were quantified after 30 minutes of incubation at room temperature in a Beckton Dickinson BD 3096 luminometer.

### AnexinV/PI staining

Cells were treated with Sorafenib 10mM or equivalent amount of DMSO for 16 hours. Media were discarded and cells were trypsinized and collected by centrifugation at 500 g, 3 min, 4°C. Pellets were washed twice with cold PBS, resuspended in Annexin V buffer, and stained with Annexin V-FITC/Propidium Iodide detection kit (Inmunostep, Salamanca, Spain) according to manufacturer’s instructions. Cells were kept for 5 minutes on ice in the dark before analysing with a MACSQuantifier 10 cytometer (Miltenyi Biotec, Bergisch Gladbach, Germany). Ten thousand cells were analysed per condition.

#### shRNA knock-down assays, lentiviral production and infections

Plasmids coding for short hairpin RNA (shRNA) against ERK5 and ATG5 were purchased from Sigma-Aldrich (SHCLNG-NM_139034 and SHCLND-NM_004849 respectively). Prior to the experiments, the best performing shRNA clone was selected as judged by Western blot using antibodies against endogenous ERK5 or ATG5.

Lentiviral production and infections were performed as previously described [23]. Briefly, HEK 293T packaging cells were cotransfected with pSAXS (helper plasmid) and pVSV-G (envelope plasmid) lentiviral vectors along with either pLKO-puro-shERK5, PLKO-puro-shATG5 or pLKO-puro empty vector (SHC001, Sigma-Aldrich) plasmids. ACHN cells were infected by adding packaging cells’ media in the presence of 4 μg/mL polybrene from Sigma-Aldrich (H9268). Forty-eight hours after infection cells were exposed to 2 μg/mL puromycin (Invivogene) and incubated for at least 3 days before any assay was undertaken. Infected cells were routinely maintained at the appropriate concentrations of puromycin.

### Western blotting assays

Western blotting detection was performed as previously described [24] Briefly, sub-confluent monolayer cultures were collected in lysis buffer (100 mM HEPES, pH = 7.5, 50 mM NaCl, 0.1% Triton X-100, 5 mM EDTA, 0.125 M EGTA). Protease and phosphatase inhibitors (0.2 mg/mL Leupeptin, 2 mg/mL, Aprotinin, 1mM PMSF and 0.1mM Na_3_VO_4_) were added prior to lysis. Indicated amounts of protein were separated on 10–12% SDS-PAGE gels, transferred to PVDF membranes (IPVH00010, Millipore), and immunoblotted using antibodies against specific proteins. Protein quantification was performed using the BCA Protein Assay Kit (Pierce, Madrid, Spain) following the manufacturer’s instructions. Results show a representative blot out of three with comparable results.

### Q-RT-PCR

RNA from was extracted with RNeasy Mini Kit (Qiagen). cDNA synthesis was performed with RevertAid First Strand cDNA synthesis Kit (Thermo Scientific) following the manufacturer’s protocol. Real-time PCR was performed with Fast SYBR Green Master kit (Thermo Scientific) in a 7500 Fast Real-Time PCR instrument (Applied Biosystems). Primer sequences used are as follows: p62/SQSTM1 F: 5’-CAGTCCCTACAGATGCCAGA-3’, p62/SQSTM1 RV: 5’-TCTGGGAGAGGGACTCAATC-3’; GAPDH F: 5’-TCGTGGAAGGACTCATGACCA-3’; GAPDH RV: 5’-CAGTCTTCTGGGTGGCAGTGA-3.

### Data analysis

Results are represented as the mean ± standard deviation of at least three independent experiments. Statistical analysis was performed using the GraphPad Prism 5.00 software. Significance was determined using a t-test. The statistical significance of differences is indicated in figures by asterisks as follows: * ⇒ p < 0.05, ** ⇒ p < 0.01 and *** ⇒ p < 0.001.

## Results

### Sorafenib inhibits ERK1/2 but not p38 MAPK in sensitive cells

**Fig 1:**
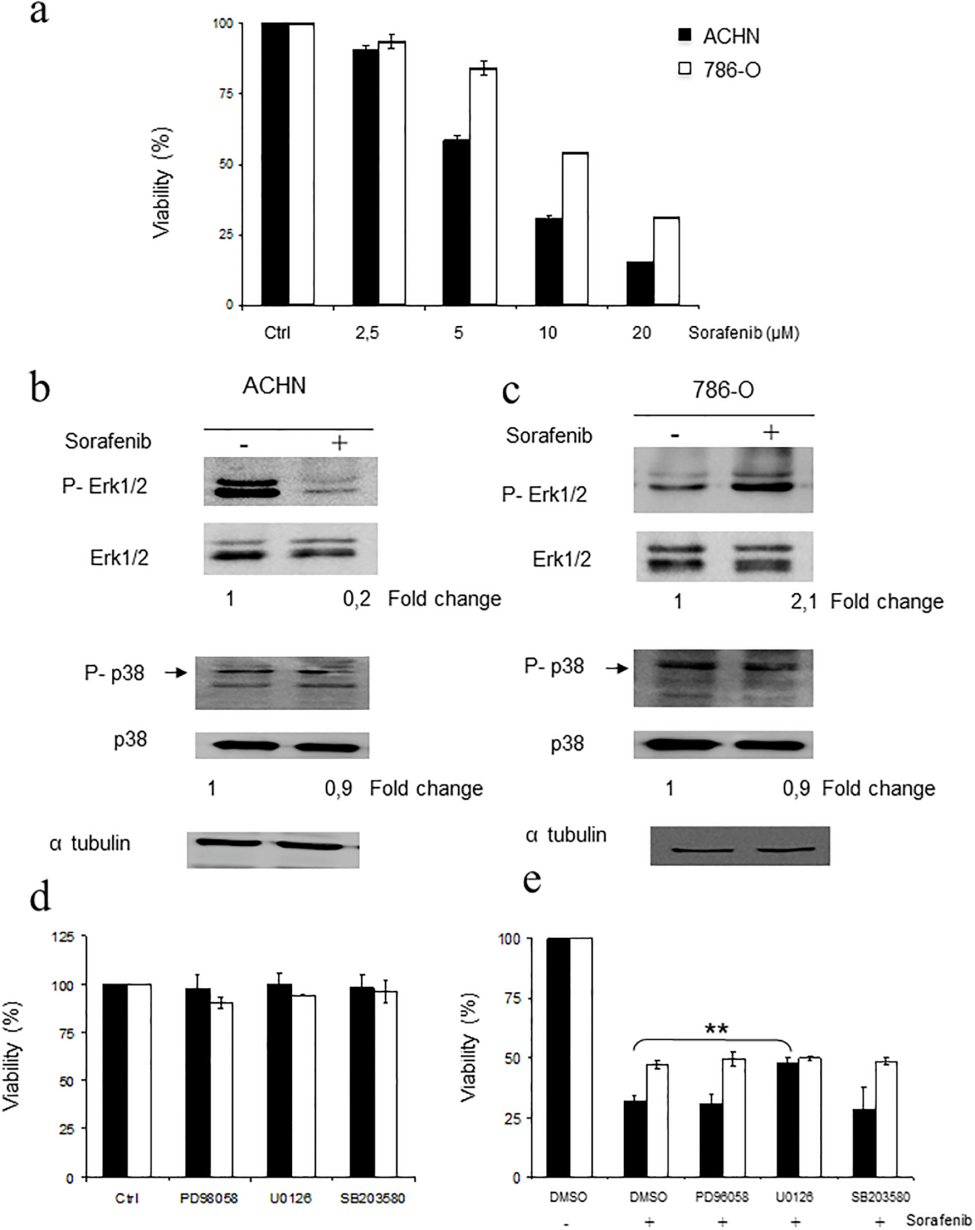
Sorafenib toxicity is not related to MAPK. a) ACHN and 786-O cells were treated for 48 h at the indicated concentrations of Sorafenib and viability was assessed by MTT assay. Black bars indicate ACHN and white bars indicate 786-O. b) ACHN and c) 786-O cells were exposed to 10 μM Sorafenib for 16 hours. Protein extracts were blotted against with the indicated antibodies. Tubulin was used as a loading control. d) Cells were treated for 48 h with 10 μM U0126, 10 μM PD98059 or 10 μM SB203580. Viability was measured by MTT assay. Black bars indicate ACHN and white bars indicate 786-O. e) ACHN and 786-O cells were treated with Sorafenib (10 μM) in combination with the indicated inhibitors (10 μM each) for 48 h. Viability was measured by MTT assay. Black bars indicate ACHN and white bars indicate 786-O. Densitometric quantification of the signals on the Western blots is shown for each picture (fold active/total) below each group.

First, we evaluated the toxicity mediated by Sorafenib in our experimental model of ACHN and 786-O cells lines of RCC. As shown in Fig 1A, cell viability analysed by MTT assay decreased in a dose-dependent fashion in both cell lines. However, 786-O cells displayed a marked resistance to Sorafenib compared to ACHN cells (IC50 of 11,15 and 6 μM respectively, p < 0.01). Next, the role of the ERK1/2 signalling pathway in the response to Sorafenib was evaluated in both cell lines, considering the potential role of Sorafenib as a Raf inhibitor. As shown in Fig 1B, a marked decrease in ERK1/2 phosphorylation was observed in ACHN cells in the presence of Sorafenib, whereas a different MAPK, p38 MAPK, remained almost unaffected. Inhibition of ERK1/2 was already detectable after 2 hours S1 Fig. However, in the case of 786-O cells, no effect was detected for any of the MAPK analysed. In fact, even an increase in ERK1/2 phosphorylation, one of the hallmarks of resistance to RAF inhibitors (see [25], could be observed (Fig 1C). These data suggest an apparent correlation between ERK1/2 inhibition and sensitivity to Sorafenib. Therefore, we decided to challenge the role of ERK1/2 in the toxic effect of Sorafenib using PD98059 and U0126, both specific inhibitors of MEK1/2 [26,27]. Interestingly, no effect on cell viability was observed by the presence of the MEK1/2 inhibitors for both cells lines (Fig 1D). Functionality of MEK1/2 inhibitors was verified by Western blotting S2 Fig. Furthermore, another MAPK, ERK5, that is related to ERK1/2, and is also inhibited by U0126 and PD98059 [28], was studied as well, showing no effect in the response to Sorafenib as indicated by genetic interference S3 Fig. The role of p38 MAPK was also evaluated using the specific inhibitor SB203580 [29], again showing no effect in terms of viability (Fig 1D). Finally, a combination of the aforementioned MAPK inhibitors with Sorafenib was studied in both cell lines, showing no effect in cell viability, except for U0126 in ACHN cells, which showed a protective effect (Fig 1E).

Therefore, these data clearly indicate that the MAPKs analysed are not key players in the toxic effect of Sorafenib in RCC cell lines.

### Sorafenib toxic effects in RCC-derived cell lines are mediated through the Akt signalling pathway

**Fig 2:**
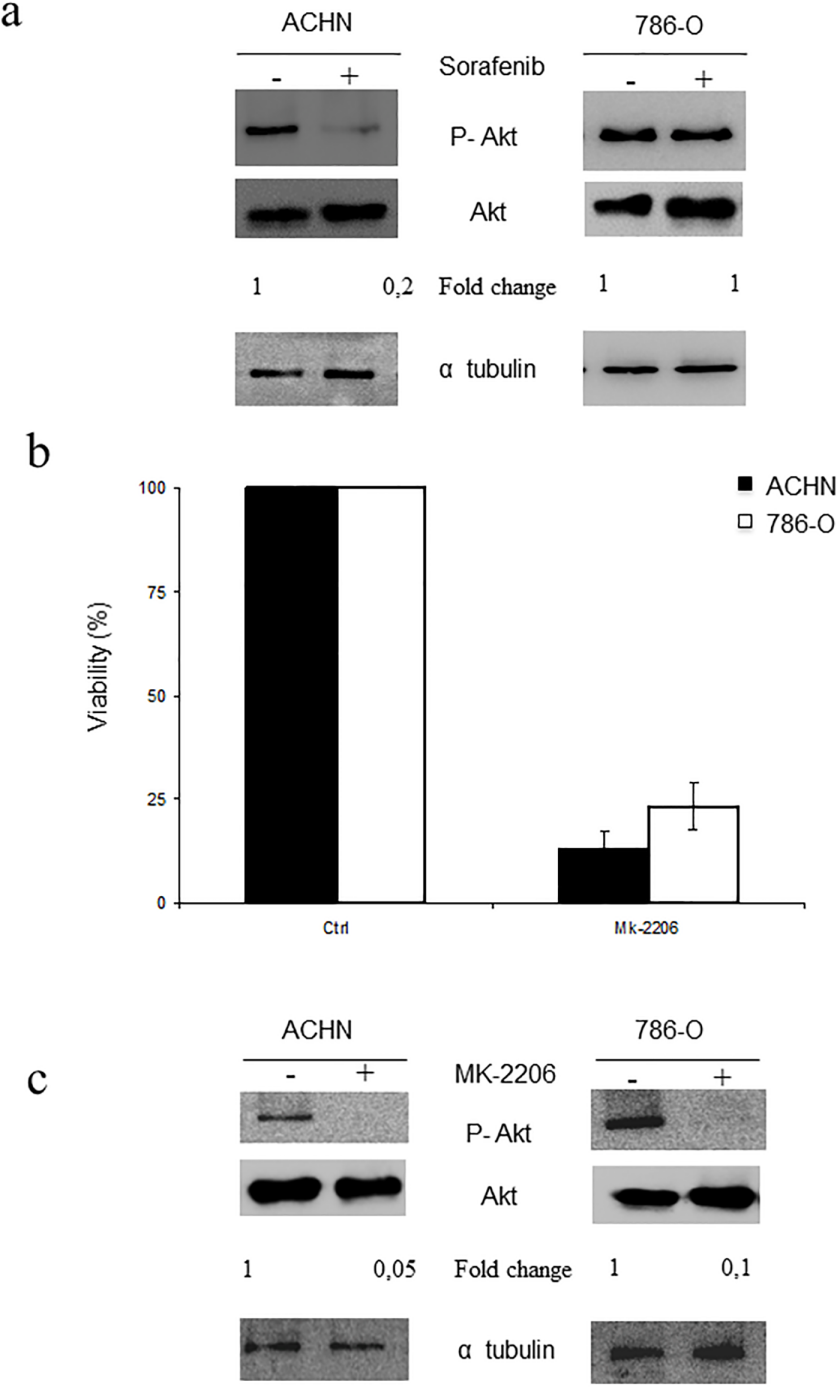
Akt is a major determinant of the toxicity associated to Sorafenib. a) Cells were treated with 10 μM Sorafenib for 16 h and protein extracts were blotted with the indicated antibodies. Tubulin was used as a loading control. b) ACHN and 786-O cell lines were treated with 10 μM MK-2206 for 48 h. Viability was assessed by MTT assay. Black bars indicate ACHN and white bars indicate 786-O. c) ACHN and 786-O cells were treated with 10 μM MK-2206 for 16 hours and protein extracts were blotted with the indicated antibodies. Tubulin was used as a loading control. Densitometric quantification of signals on the Western blots is shown for each picture (fold active/total) below each group.

To further investigate the molecular basis of Sorafenib-associated toxicity in RCC, we decided to challenge another signalling pathway known to be implicated in Sorafenib response, the PI3K/Akt signalling pathway [30]. As shown in Fig 2A, a marked decrease in Akt serine 473 phosphorylation was observed in the presence of Sorafenib in ACHN but not in 786-O cells (Fig 2A), again showing a strong correlation with sensitivity. To fully evaluate the role of Akt, we used a specific Akt inhibitor, MK-2206 [31], which exhibited a marked toxicity in both experimental models (Fig 2B), Functionality of MK-2206 was evaluated in both experimental models showing a potent inhibition of Akt activation (Fig 2C).

Therefore, our data suggest a role for the Akt signalling pathway in the toxic effect of Sorafenib, discarding a direct participation of ERK1/2.

### Akt inhibition and Sorafenib promotes autophagic cell death without caspase activation

**Fig 3:**
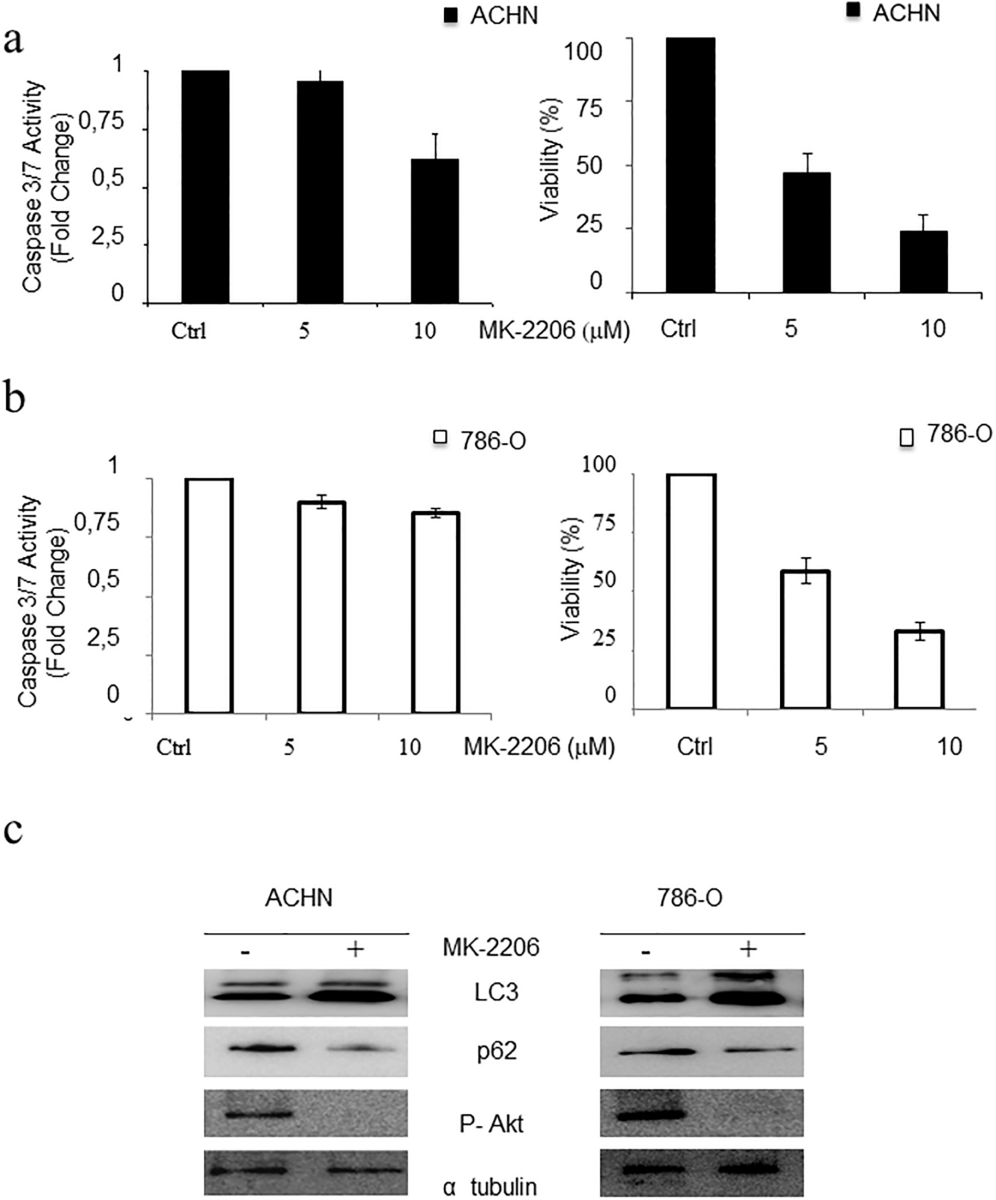
Autophagy mediates cells death associated to Akt inhibition. a) ACHN cells were treated with MK-2206 at the indicated concentrations (μM) for 24 hours and caspase 3/7 activity was evaluated (Left panel). Cell viability was evaluated under the same conditions by MTT assay (Right panel). b) Caspase 3/7 activity (Left panel) and cell viability (right panel) were evaluated in 786-O cells as indicated in A). c) Cells were exposed to 10 μM MK-2206 for 16 hours. Protein extracts were blotted with the indicated antibodies. Tubulin was used as a loading control.

**Fig 4:**
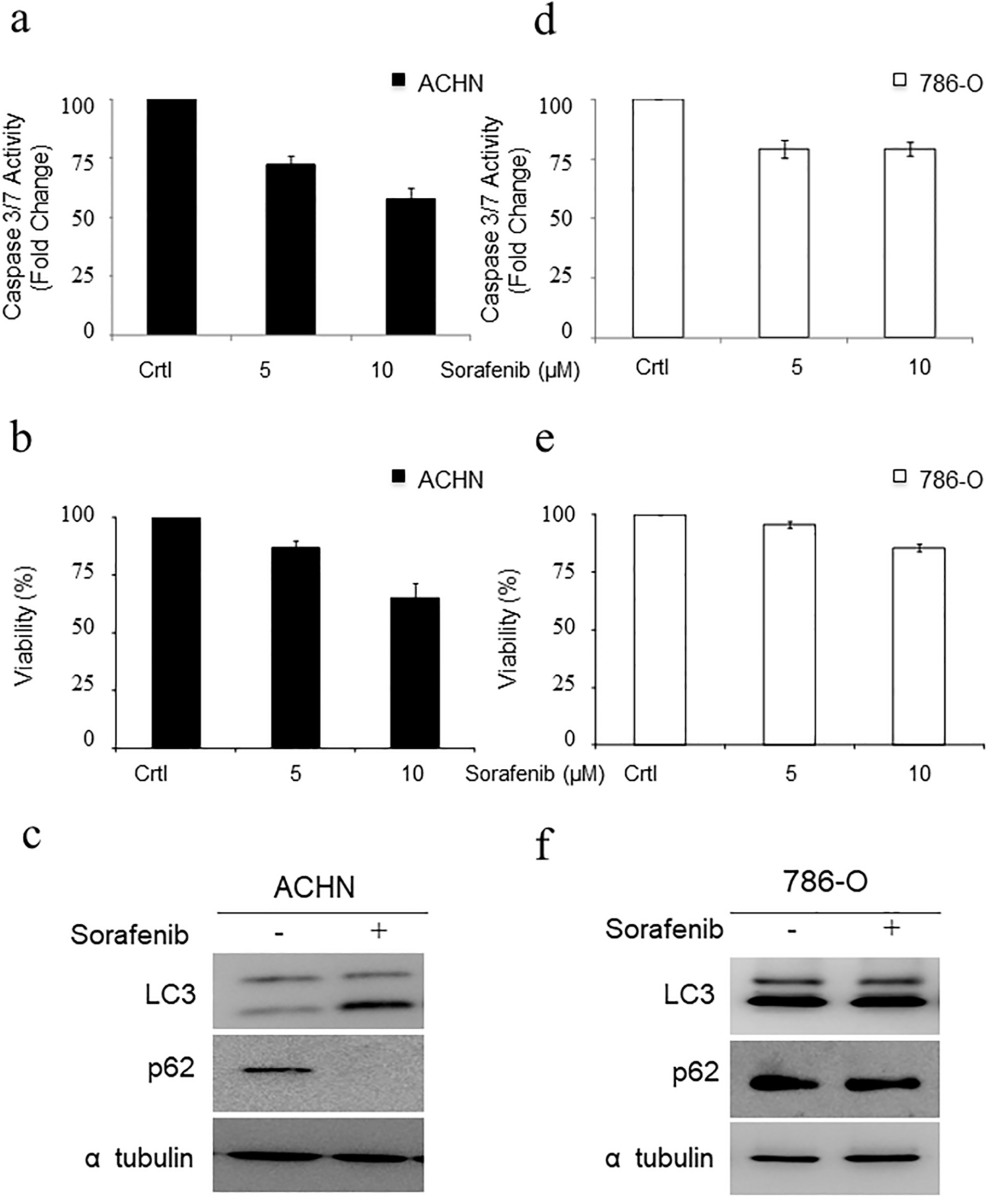
Autophagy is the main mechanism of cell death associated to Sorafenib. a) Caspase 3/7 activity was evaluated in ACHN cells treated with Sorafenib for 24 hours. b) Viability of ACHN cells was evaluated under the same conditions by MTT assay. c) ACHN cells were exposed to 10 μM Sorafenib for 16 hours. Protein extracts were blotted with the indicated antibodies. d) Caspase 3/7 activity was evaluated in 786-O cells treated with Sorafenib for 24 hours. e) Viability of 786-O cells was evaluated by MTT assay as in B). f) 786-O cells were exposed to 10 μM Sorafenib for 16 hours. Protein extracts were blotted with the indicated antibodies. Tubulin was used as a loading control.

In light of this previous finding, we analysed the molecular mechanism of cell death associated to Sorafenib. First, induction of caspases 3/7 was evaluated in ACHN and 786-O cells exposed to MK-2206. Neither of the two cell lines showed caspase 3/7 activation (Fig 3A and 3B). In contrary, a slight decrease in caspase 3/7 activation was observed, probably due to the toxicity associated with MK-2206 treatment (Fig 3A and 3B, right panels). Next, we evaluated the induction of autophagy in these cells by means of LC3 lipidation and p62/SQTM1 degradation [32]. Both cell lines showed a LC3 lipidation and p62 degradation pattern in response to Akt inhibition that is consistent with induction of autophagy (Fig 3C), Next, the cell death mechanism associated to Sorafenib was studied by evaluating caspase 3/7 activity in ACHN cells in response to Sorafenib. No activation of caspase 3/7 was detected (Fig 4A), but instead we observed a decrease that correlates with the expected low toxicity of the drug (Fig 4B). Furthermore, AnexinV/PI staining was analysed in response to Sorafenib in the ACHN cell line, showing a result consistent with a marginal role for conventional apoptosis in Sorafenib-associated cell death S4 Fig. We then evaluated the induction of autophagy mediated by Sorafenib in ACHN cells, detecting a clear increase in LC3 lipidation and p62 degradation (Fig 4C). To fully confirm that p62 degradation was not due to a transcriptional regulation, q-RT-PCR assays were performed, showing no alteration in mRNA levels due to the presence of Sorafenib S5 Fig. This indicates that the observed changes in p62 protein levels were due to post-translational modifications rather than alterations in gene expression. Furthermore, a marked decrease in Akt dependent phosphorylation of m-TOR (ser 2448) was observed in response to Sorafenib S6 Fig, consistent with the induction of autophagy.

**Fig 5:**
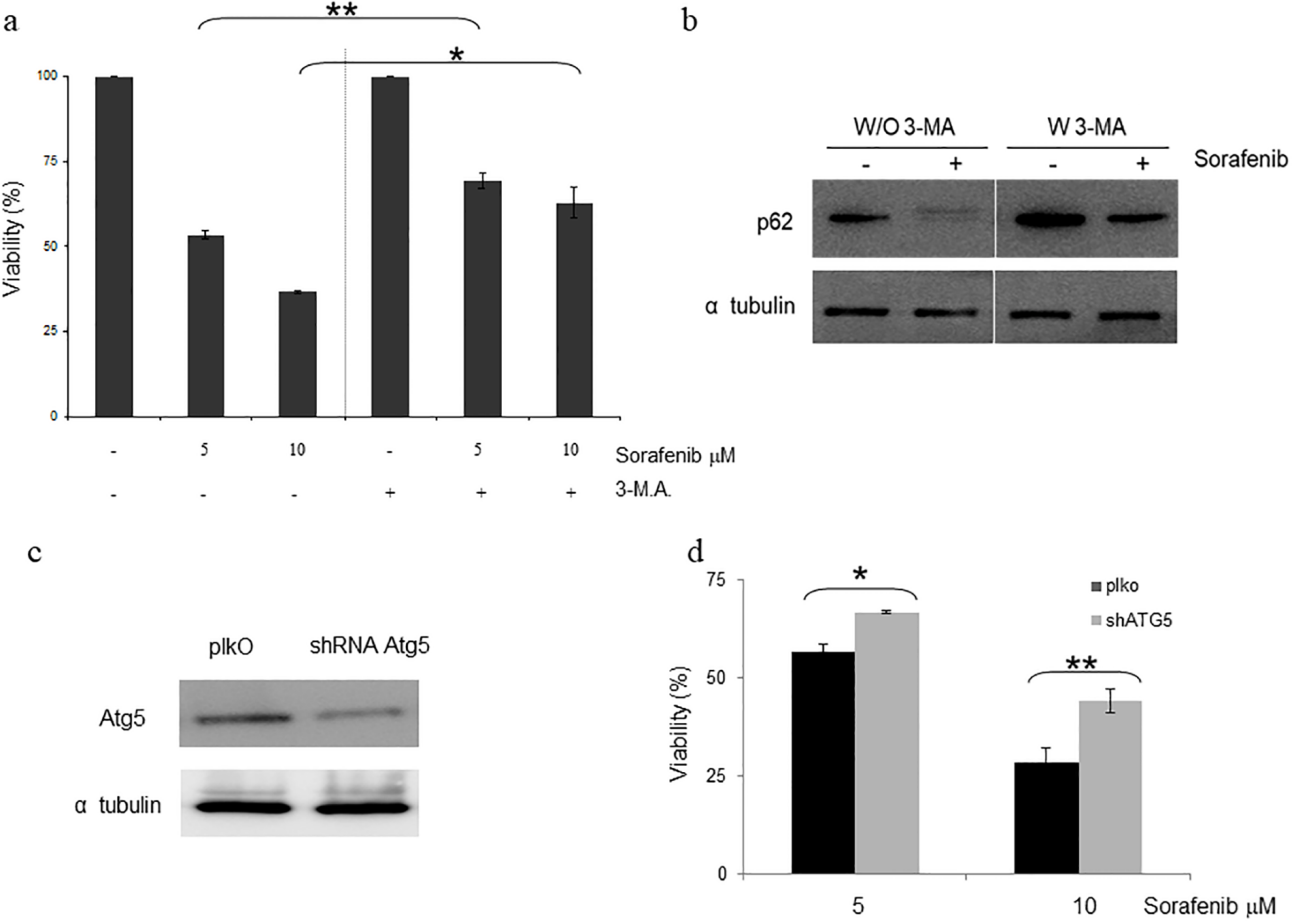
Blockage of autophagy promotes resistance to Sorafenib in ACHN cells. a)ACHN cells were treated with Sorafenib at the indicated concentrations in the presence or absence of 2.5 mM 3-M.A. Viability was assessed by MTT assay after 48 hours. b) ACHN cells were treated with Sorafenib at 10 μM in the presence or absence of 2.5 mM 3-M.A for 16 hours. The protein extracts were collected and blotted with the indicated antibodies. Tubulin was used as a loading control. c) ACHN cells were infected with lentivirus carrying empty vector or a specific shRNA against ATG5. Selected pools were lysed and protein extracts were blotted with the indicated antibodies. Tubulin was used as a loading control. d) ACHN cells that were infected with lentivirus carrying empty vector (black bars) or a specific shRNA against ATG5 (grey bars) were treated with Sorafenib for 48 hours at the indicated concentrations and viability was assessed by MTT assay.

Finally, in the experimental model of 786-O cells no caspase 3/7 activation (Fig 4D), positivity for AnexinV staining S4 Fig, LC3 lipidation or p62 degradation (Fig 4F) was observed, consistent with the marked resistance to Sorafenib observed in this cell line (Fig 4E). To fully prove the direct implication of autophagy in the cellular response to Sorafenib, we used the autophagy specific inhibitor 3-MA [33]. As shown in Fig 5A, 3-MA promotes an increase in the resistance to Sorafenib that correlates with a blockage of autophagy (Fig 5B). To support our observation that is based on chemical approaches, genetic interference of ATG5, a key gene in autophagy [34], was performed (Fig 5C). As shown in Fig 5D, lack of ATG5 also renders a pattern of resistance. Therefore, our data support that Sorafenib executed its toxic effect through the induction of an autophagic response.

To sum up, this set of experiments clearly indicates that autophagy is the main mechanism of cell death associated with Sorafenib in our experimental model of RCC.

## Discussion

Several conclusions can be obtained from the present report.

The first observation is about the role of ERK1/2 signalling pathway in the response of RCC-derived cell lines to Sorafenib. Our data indicate that the activity of Sorafenib, as a Raf inhibitor, is not implicated in the therapeutic effects of Sorafenib in RCC. This finding supports clinical data that suggest the use of this tyrosine kinase inhibitor based on its activity towards VEGF-R and PDG-R rather than its ability as a Raf inhibitor, consistent with previous reports [35,36]. In fact, it is notorious that the inhibition of the ERK1/2 signalling pathway did not affect the viability of RCC-derived cell lines, explaining previous observations such as those with the 786-O xenograft model [37]. Interestingly, we observed a moderate increase in ERK1/2 activity in 786-O cells in the presence of Sorafenib that correlated with drug resistance. This observation is not new as it has been observed in a different experimental model [38–41]. A number of different mechanisms have been proposed that could account for the lack of inhibition and Sorafenib resistance, such as mutation in Ras genes [42], elevated levels of RAF [43], or overexpression of COT [44]. Nonetheless, the specific case of 786–0 cells needs to be fully addressed. In any case, our data discard the ERK1/2 signalling pathway as a primary target in RCC therapy, excluding the use of novel MEK/ERK inhibitors [45]. However, lack of implication in the toxic effect of Sorafenib does not exclude the ERK1/2 signalling pathway in terms of resistance. We observed a marked correlation between sensitivity to Sorafenib and ERK1/2 inhibition in our model of RCC. Consistent with this, an apparent role of this signalling pathway in Sorafenib resistance in hepatocellular carcinoma [16], and also in a RCC mouse-derived cell line resistant by co-culturing [21] has been reported. However, it has been described that combination of ERK1/2 inhibitors potentiates the therapeutic effect of low doses of Sorafenib in hepatocellular carcinoma [46], whereas in our experimental model we only observed a protective effect with one of the ERK1/2 signalling pathway inhibitors. The apparent contradictory results between the used ERK1/2 inhibitors could be explained, in part, by the fact that UO126 is known to be a more promiscuous inhibitor. For example, its effect on other MAPK pathways in addition to ERK1/2 [28] as well as on some of the downstream effectors of the Pi3K/Akt/ mTOR signalling pathway as p70S6K have been described [47]. Taken together, all these data suggest that, although in an indirect fashion, ERK1/2 might also be implicated in some aspects of the Sorafenib “acquired resistance” machinery but not in the “*de novo*” response. Nonetheless, further studies are necessary to fully establish the role of ERK1/2 in Sorafenib resistance in RCC, as well as in other experimental models.

Second, regarding to p38 MAPK, it is noteworthy that based on the lack of an effect mediated by Sorafenib on p38 MAPK activation as well as the lack of an effect of SB203580, alone or in combination with Sorafenib, our data do not support a direct implication of this signalling pathway in the response of RCC-derived cell lines to Sorafenib treatment. However, recent studies have shown that p38 MAPK mediates resistance to Sorafenib *in vivo* in hepatocellular carcinoma (HCC) [48]. This apparent contradiction with our results could be explained by the fact that our observations are obtained in cell culture, not *in vivo*, as well as by the different pathologies studied (RCC versus HCC). Nonetheless, the lack of implication of p38 MAPK has already been established in other experimental models such as chondrosarcoma [49], suggesting that p38 MAPK effects in Sorafenib could be cell type specific.

The third issue is the mechanism of cell death associated with Sorafenib. In our experimental model of RCC, cell death is a caspase-independent process, which is in agreement with previous observations in other experimental settings, such as in Melanoma [50]. Interestingly, in other experimental models, caspase-dependent and -independent mechanisms seem to be coexisting, for example, in Multiple Myeloma [51]. However, in other cases, as in Mantle Cell Lymphoma or some types of Leukaemia, sorafenib-induced cell death is mediated by apoptosis. [52,53]. Therefore, considering our data, the use of proapoptotic drugs, like the newly developed BH3 mimetic [54], independent of a putative synergic/additive effect as has been reported in glioblastoma, will not be the best option to promote sensitivity to Sorafenib in RCC patients [55]. Furthermore, recent evidences involve autophagy as a key player in the cellular response to Sorafenib with therapeutic repercussion (for review see [56,57]). There is evidence supporting that autophagy is the main mechanism of cell death associated with Sorafenib in different experimental models [58,59], and that it has a direct effect on key molecules in the control of autophagy such as m-TOR and downstream targets [60,61], supporting our observation on RCC. Interestingly, other studies in Hepatocellular Carcinoma or Glioblastoma suggest a protective effect for autophagy, based on how inhibition of autophagy potentiates the effectiveness of Sorafenib [62,63]. However, our data indicates that autophagy seems to be the main mechanism of cell death associated with Sorafenib and a key component of the *de novo* resistance in RCC. In addition, our observations seem to fit perfectly with recent studies proposing that Akt inhibition in response to Sorafenib switches autophagy from a cytoprotective to a cytotoxic mechanism [64]. Therefore, considering the dual role of autophagy in cancer therapy [65] as well as the particular behaviour of RCC models in the pharmacological control of autophagy [66], promotion of autophagy in RCC therapy could be a new therapeutic window for patients refractory to Sorafenib that needs to be carefully evaluated.

Finally, it is noteworthy the role of Akt inhibition. This effect is clearly inferred from the data obtained with the Akt inhibitor MK-2206. The fact that this Akt inhibitor mediates cell death in a similar way as Sorafenib, even in resistant cells like the 786-O line, provides strong support for the use of new PI3K/Akt/mTOR inhibitors in the treatment of RCC (for a review see [67]). Furthermore, our data support a recent observation suggesting that Akt1 interference promotes sensitivity to Sorafenib in ACHN cells [68]. However, several differences between the two studies should be considered. For example, this previous work is based on shRNA approaches that indicate a correlation between Akt1 expression levels and Sorafenib response, but no direct cause-effect mechanism is established. Moreover, the previous study did not analyse any autophagic parameter nor showed any *in vitro* biochemical evidence for apoptosis. Nonetheless, the use of Akt inhibitors in the treatment of cancer is clearly a new therapeutic window owing to the effect exerted in apoptosis or autophagy that could be cell-type dependent [31,69].

In summary, we present data supporting that Sorafenib mediates its toxic effect in RCC-derived cell lines through the induction of autophagy triggered by the inhibition of Akt in a MAPK-independent manner. Whether our proposed mechanism could apply to other types of tumors and putative implications in the therapy of RCC needs to be further investigated.

## Supporting information

S1 Fig ACHN cells were exposed to 10 μM Sorafenib for the indicated time points and 50 μg of protein extracts were blotted with the indicated antibodies. Tubulin was used as a loading control.
(TIF)

S2 Fig a) ACHN cells were exposed to 10 μM U0126 for 16 hours and protein extracts were blotted with the indicated antibodies. b) ACHN cells lines were exposed to 10 μM PD98059 for 16 hours. Fifty μg of protein extracts were blotted with the indicated antibodies. Tubulin was used as a loading control.
(TIF)

S3 Fig a) Proetin extracts (100 μg) of ACHN cells infected with lentivirus carrying an empty vector or an shRNA against ERK5 were blotted against ERK5. b) ACHN cells carrying an empty vector or shRNA against ERK5 were treated with 5 or 10 μM of Sorafenib for 48Hours and cell viability was measured by MTT assay. Black bars indicate empty pLKO vector and grey bars indicate shERK5 vector.
(TIF)

S4 Fig ACHN and 786–0 cells were treated with Sorafenib 10 μM for 16h and positivity for Annexin V-FITC/Propidium Iodide was evaluated in a MACSQuantifier 10 cytometer (Miltenyi Biotec, Bergisch Gladbach, Germany). Ten thousand cells were analysed per condition.
(TIF)

S5 Fig Analysis of p62 mRNA expression levels in ACHN cells treated with Sorafenib (10 μM) or Rapamycin (200mM) for 16 hours. Expression levels were calculated using 2 ^-ΔΔCt^ method using GAPDH expression as a reference and values were referred to non-treated cells. Results are shown as mean±SD.
(TIF)

S6 Fig ACHN cells were exposed to 10 μM Sorafenib or 200 nM Rapamycin for 16 hours. Protein extracts (100 μg) were blotted against indicated antibodies. Vinculin was used a as a loading control.
(TIF)
