# Supplementary figures and images for: Autophagic cell death associated to Sorafenib in renal cell carcinoma is mediated through Akt inhibition in an ERK1/2 independent fashion
Source: PLoS One. 2018 Jul 26;13(7):e0200878. doi: 10.1371/journal.pone.0200878 (PMC6062059; doi:10.1371/journal.pone.0200878)

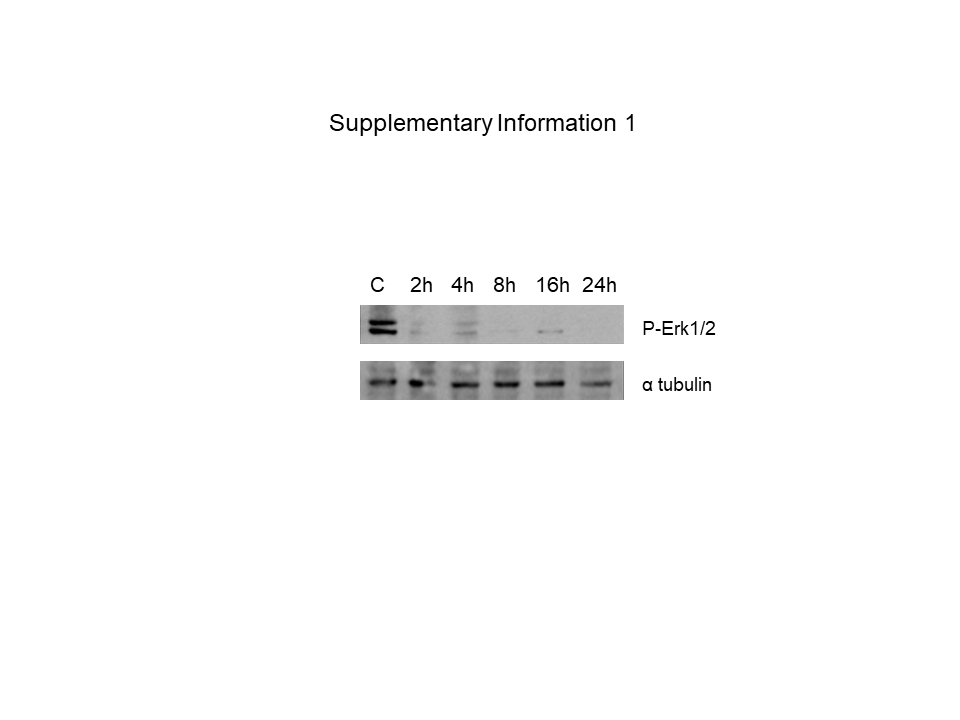

Supplement: S1 Fig — Tubulin was used as a loading control. (TIF) [file pone.0200878.s001.tif]

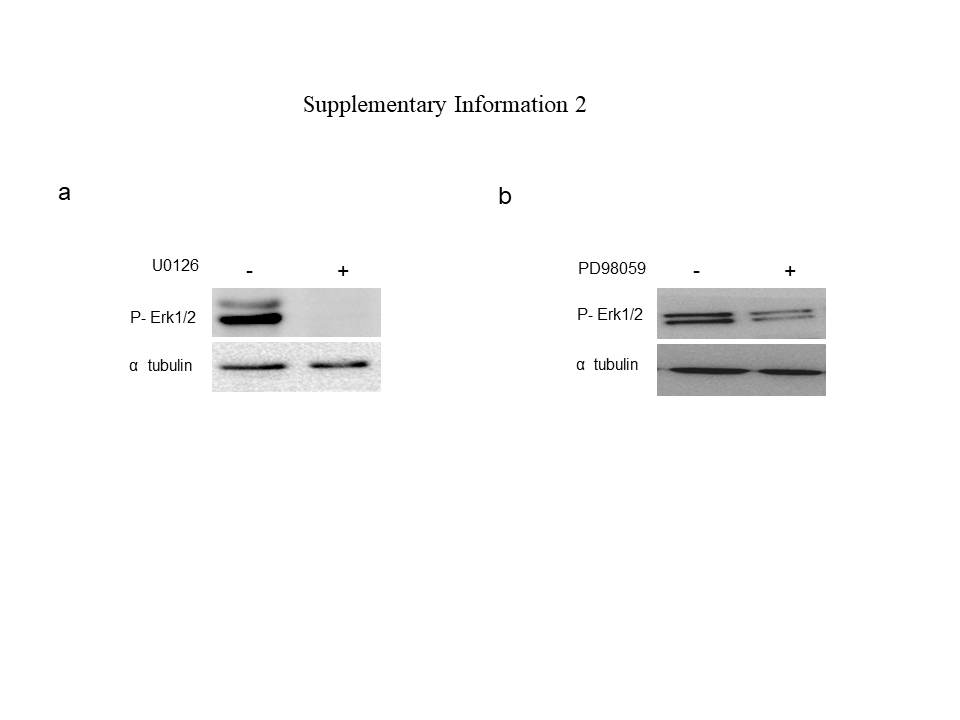

Supplement: S2 Fig — a) ACHN cells were exposed to 10 μM U0126 for 16 hours and protein extracts were blotted with the indicated antibodies. b) ACHN cells lines were exposed to 10 μM PD98059 for 16 hours. Fifty μg of protein extracts were blotted with the indicated antibodies. Tubulin was used as a loading control. (TIF) [file pone.0200878.s002.tif]

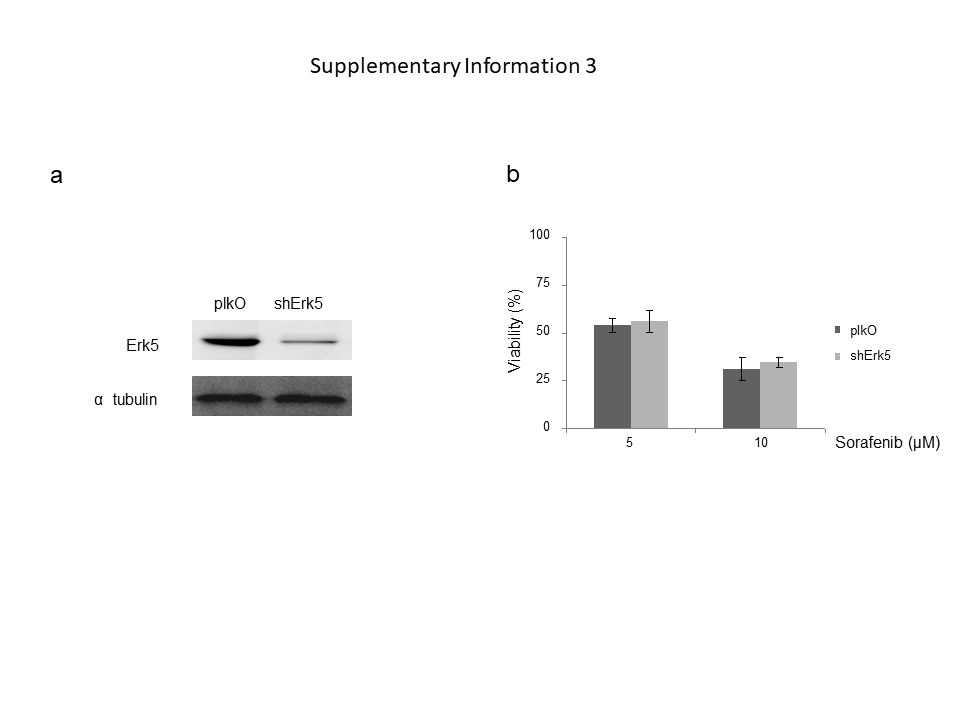

Supplement: S3 Fig — a) Proetin extracts (100 μg) of ACHN cells infected with lentivirus carrying an empty vector or an shRNA against ERK5 were blotted against ERK5. b) ACHN cells carrying an empty vector or shRNA against ERK5 were treated with 5 or 10 μM of Sorafenib for 48Hours and cell viability was measured by MTT assay. Black bars indicate empty pLKO vector and grey bars indicate shERK5 vector. (TIF) [file pone.0200878.s003.tif]

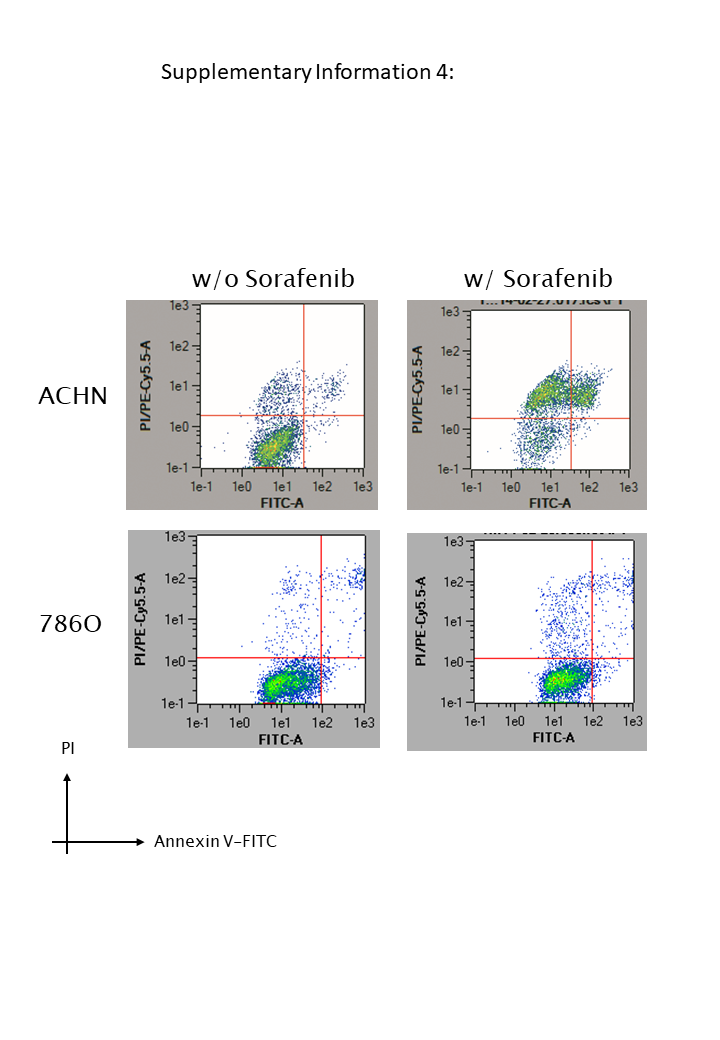

Supplement: S4 Fig — Ten thousand cells were analysed per condition. (TIF) [file pone.0200878.s004.tif]

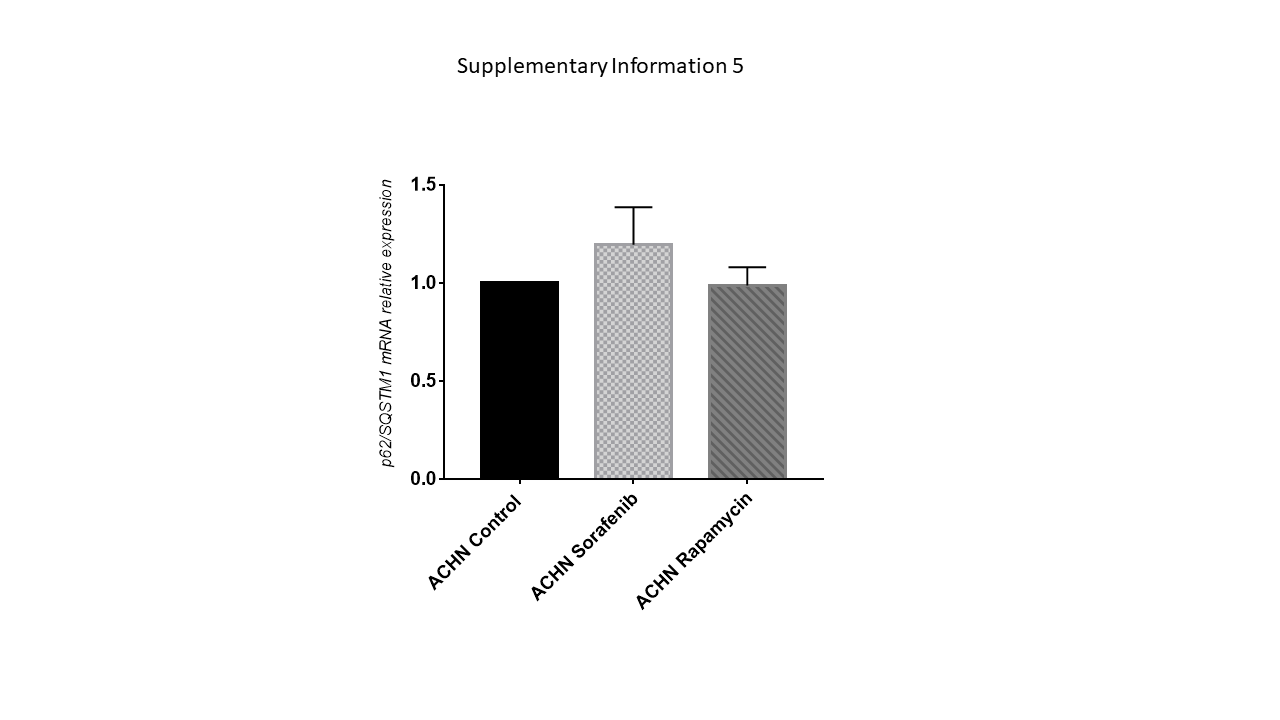

Supplement: S5 Fig — Expression levels were calculated using 2 -ΔΔCt method using GAPDH expression as a reference and values were referred to non-treated cells. Results are shown as mean±SD. (TIF) [file pone.0200878.s005.tif]

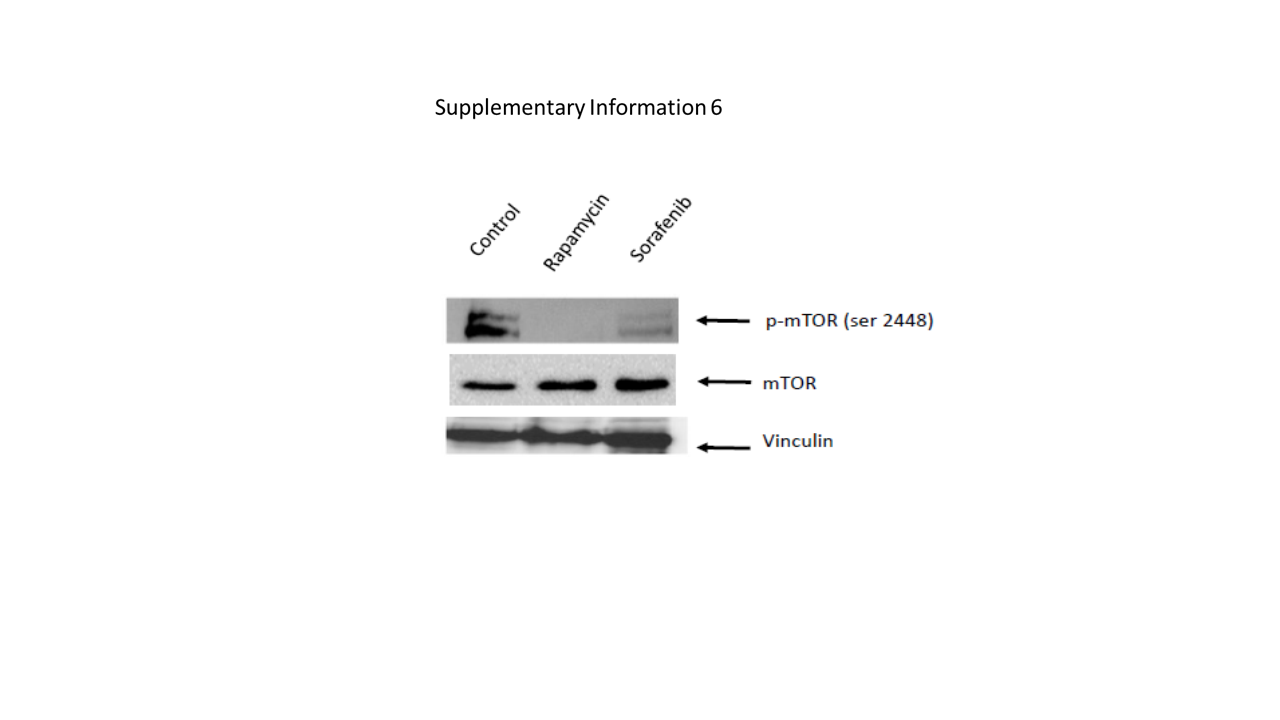

Supplement: S6 Fig — Protein extracts (100 μg) were blotted against indicated antibodies. Vinculin was used a as a loading control. (TIF) [file pone.0200878.s006.tif]
